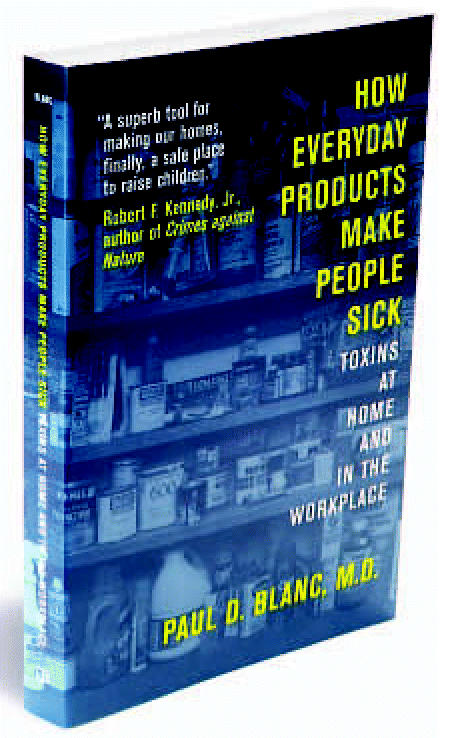# How Everyday Products Make People Sick: Toxins at Home and in the Workplace

**Published:** 2007-03

**Authors:** Howard M. Kipen

**Affiliations:** Howard M. Kipen directs the Clinical Research and Occupational Medicine Division at the Environmental and Occupational Health Sciences Institute of UMDNJ-Robert Wood Johnson Medical School, where he is a professor. He does controlled exposure studies on air pollutants

By Paul D. Blanc

Berkeley: University of California Press, 2007. 374 pp. ISBN: 0-520-24882-1, $19.95

Most individuals who are concerned with public health, specifically occupational and environmental health, have some awareness that the field did not begin with Rachel Carson, the London Fog, Irving Selikoff, or fevers from welding galvanized steel. In seven compelling chapters on the development, recognition, and unfortunately re-recognition of occupational and environmental disease, Paul Blanc, treats readers to a marvelous distillation of the side effects and misadventures of industrial development over the last 300 years, arguing that workers and consumers would be healthier if we learned from our past. Although the exact prescriptive balance between innovation and regulation is not, and probably could not be, explicitly presented, the book triumphs in its thorough explication of how so many modern technologies and their accompanying maladies developed, evolved, and redeveloped. Blanc deftly presents uncommon histories about common environmental agents, and weaves connections at the medical and industrial levels that provide glue for the bare facts.

The chapter “Good Glue, Better Glue, Superglue” teaches us traditional glue making from animal collagen, and then detours to the development of magenta and other dyes from coal tar distillation, producing unwanted benzene. Blanc then describes the use of benzene as a solvent for rubber cement and its attendant effects on the bone marrow, and polymer sealants such as nitrocellulose dissolved in tetrachloroethane when benzene was diverted to munitions manufacture during World War I. Subsequently he describes the development and medical consequences of celluloid, plastic polymers such as Bakelite, polyvinyl chloride manufacture, toluene, hexane, acetontrile and artificial fingernails, and finally isocyanate adhesives. Along the way, the inadequacy of health regulation is discussed, as the Consumer Products Safety Commission receives particular attention for failing to act. And this is only the “glue” chapter. Other intriguing chapters detail the 18th-century origins of chlorine bleaching and chlorine’s use in industry and as a war gas, as well as carbon disulfide’s use in making synthetic silk (rayon) and vulcanizing rubber. “Job Fever” examines how the recognition of mill fevers in 18th-century England led to the recognition of byssinosis, now attributed to endotoxin exposure. From there Blanc makes the pathophysiologic (cytokine-mediated systemic inflammation) link to metal fume fevers, going back to ancient China for the origins of brass founding. “Emerging Toxins” discusses recently recognized causes of bronchiolitis obliterans (nylon flock, popcorn flavoring) and the effects of various wood treatments (mercuric chloride, creosote, chlorinated hydrocarbons, and copper–chromium arsenate). This chapter also discusses the gasoline additive organic lead and the currently contentious use of the organic manganese compound methylcyclopentadienyl manganese tricarbonyl as its replacement, despite concern for an association of manganese with Parkinson and other neurologic diseases. Briefer treatment is given to misadventures such as the manganese-based laundry bleach developed in Europe for cold-water washing that ate through clothes when used in predominantly hot-water U.S. washing machines. However, this book keeps its eye on human health effects, which are clearly presented for a nonmedical audience, the subtleties of organ dysfunction not being the focus of this extensively footnoted history.

Readers are treated to contextual anecdotes about the players in many of these dramas, ranging from those who developed the technologies (e.g., Michael Faraday for mercuric chloride), to those who studied the conditions (e.g., Jean-Martin Charcot for carbon disulfide neurotoxicity), to multiple pitiable descriptions of the victims of industrial progress (e.g., Clara, the benzol worker).

This book is highly recommended to occupational/industrial toxicologists, academic toxicologists, occupational physicians, environmental health scientists, and especially to those responsible for regulation in these fields at local, state, federal, or international levels. It will prove invaluable for those who seek to contextualize, illustrate, and personalize lectures to public health, toxicology, and other students. It also provides illuminating background information on the evolution of modern life. Unfortunately for its publisher, although the book may provide bon mots for dinner table conversation and the like, it may not gain as much traction for the domestic audience as its title suggests it was aimed to attract. This is not a prescriptive manual for how to make our homes safer. However, it does make a strong case that we need to improve our occupational, consumer, and environmental regulation, so that new technologies and new applications of older technologies are less often developed at the expense of health.

## Figures and Tables

**Figure:**